# Consequences of anterior knee pain after anterior cruciate ligament reconstruction: A 2015–2020 cohort study

**DOI:** 10.1371/journal.pone.0280146

**Published:** 2023-01-05

**Authors:** Marie Chantrelle, Pierre Menu, Vincent Crenn, Jérôme Grondin, Pauline Daley, Bastien Louguet, Alban Fouasson-Chailloux, Marc Dauty

**Affiliations:** 1 Service de Médecine Physique et Réadaptation Locomotrice et Respiratoire, CHU Nantes, Nantes Université, Nantes, France; 2 Département de Médecine Physique et de Réadaptation, Centre Hospitalier Universitaire d’Angers, Angers, France; 3 Service de Médecine du Sport, CHU Nantes, Nantes Université, Nantes, France; 4 Institut Régional de Médecine du Sport (IRMS), Nantes, France; 5 Inserm, UMR 1229, RMeS, Regenerative Medicine and Skeleton, ONIRIS, Nantes Université, Nantes, France; 6 Clinique Chirugicale Othopédique et Traumatologique, CHU Nantes, Nantes Université, Nantes, France; University of Hartford College of Education Nursing and Health Professions, UNITED STATES

## Abstract

Anterior cruciate ligament reconstruction (ACLR) using hamstring tendon (HT) graft aims to stabilise the knee, but it may bring some complications like anterior knee (AKP) pain that can have consequences on the functional aspect of this surgery. The aim of this study was to compare isokinetic knee strength and functional outcomes between patients with and without AKP following an ACLR using HT graft during the first-year post-surgery. Three hundred and thirty subjects operated by ACLR using hamstring tendon graft were included in our retrospective cohort and divided into two groups: a group with AKP (AKP+ group) and one without AKP (AKP–group). In our population, 14.8% of the patients had AKP. At 4 post-operative months, subjects with pain had lower isokinetic strength limb symmetry index (LSI) for knee flexors and extensors, and a lower Lysholm score than subjects without pain (p < 0.0001). These differences did not persist at 7 post-operative months, and there was no difference in the one-leg hop test. After multivariate analysis, we highlighted the impact of time on the evolution of these parameters. Yet, the exact definition of AKP after ACLR remains to be clearly defined since an imprecise diagnosis may lead to inappropriate management. Pre-operative information about this type of complication, which evolves favourably with time, could be useful for patients. Indeed, AKP can occur after ACLR, even if a HT graft has been used, compared to other surgical procedures using the knee extensor apparatus as patellar tendon graft (AKP is associated with the donor site morbidity). In case of AKP after ACLR, monitoring the muscle inhibition by isokinetic tests may enable clinicians to adapt the retraining and the return to sport.

## Introduction

Anterior cruciate ligament (ACL) tear is a frequent knee injury with an estimated prevalence of about 250 000 per year in the United States [1]. The main risk of ACL rupture is knee instability but other injuries such as meniscal or cartilage damage may be associated [2]. Thus, surgical management aims to restore knee stability by reconstructing the ACL, mainly with a hamstring tendon (HT) autograft or a patellar tendon (PT) autograft. ACL reconstruction (ACLR) is frequently associated with another surgical procedure (i.e. meniscus surgery, lateral tenodesis) [3]. The rate of ACLR is estimated to be between 60 000 and 175 000 per year in the United States, a rate that has risen sharply in the past twenty years [1,3,4].

The frequency of medical or surgical complications after ACLR is estimated from 2.3% to 39% at 2 years post-surgery [3,5]. The most common complications include infections, knee stiffness, post-traumatic osteoarthritis, difficulty to return to sport, arthrogenic muscle inhibition (AMI) and pain [1,3,6].

Among pains complicating ACLR, anterior knee pain (AKP) is a frequent complaint [1,7] which can be described as a retro- or peri-patellar pain increasing in a bent-knee position [8,9]. The medical literature reports an estimated prevalence of AKP after ACLR from 2.7% to more than 20% depending on the type of graft [5,10,11]. Indeed, AKP is less frequent in case of ACLR using a HT graft compared to ACLR using a PT graft, but it may exist in these last two conditions [2,7,12–15]. Having an ACLR using a PT graft increases the risk of developing AKP by 3.4 compared to having an ACLR using a HT graft [14]. Furthermore, AKP complicating ACLR can contribute to developing quadriceps and hamstring strength deficit corresponding to AMI [16]. In fact, both pain and surgery lead to AMI [6,17–19]. However, the literature remains poor concerning functional and objective consequences of AKP after ACLR. After PT graft procedure, kneeling appears to be the activity that generates significant AKP intensity at 3 and 6 months after ACL reconstruction [20]. Moreover, AKP is also often explained by the PT graft site morbidity. In case of HT procedure, the Kujala score and the Cincinnati Knee score are low, but these scores are used to identify only AKP defined as femoro-patellar pain [21,22]. The AMI recovery after ACLR seems to be delayed in patients with AKP compared to those without AKP [23]. Furthermore, AKP has consequences on mental health, notably concerning anxious and depressive symptoms [24]. It is also known that knee pathologies have consequences on the functional outcomes [16]. Patient-acceptable symptom state, including pain at two years post-surgery, requires a symmetrical quadricipital strength at six months post-surgery and a symmetrical jump performance at the one leg hop test [25].

The aim of this study was to compare isokinetic knee strength and functional outcomes between patients with and without AKP following an ACLR using HT graft at four and seven months post-surgery.

## Methods

### Participants and recruitment

**Fig 1:**
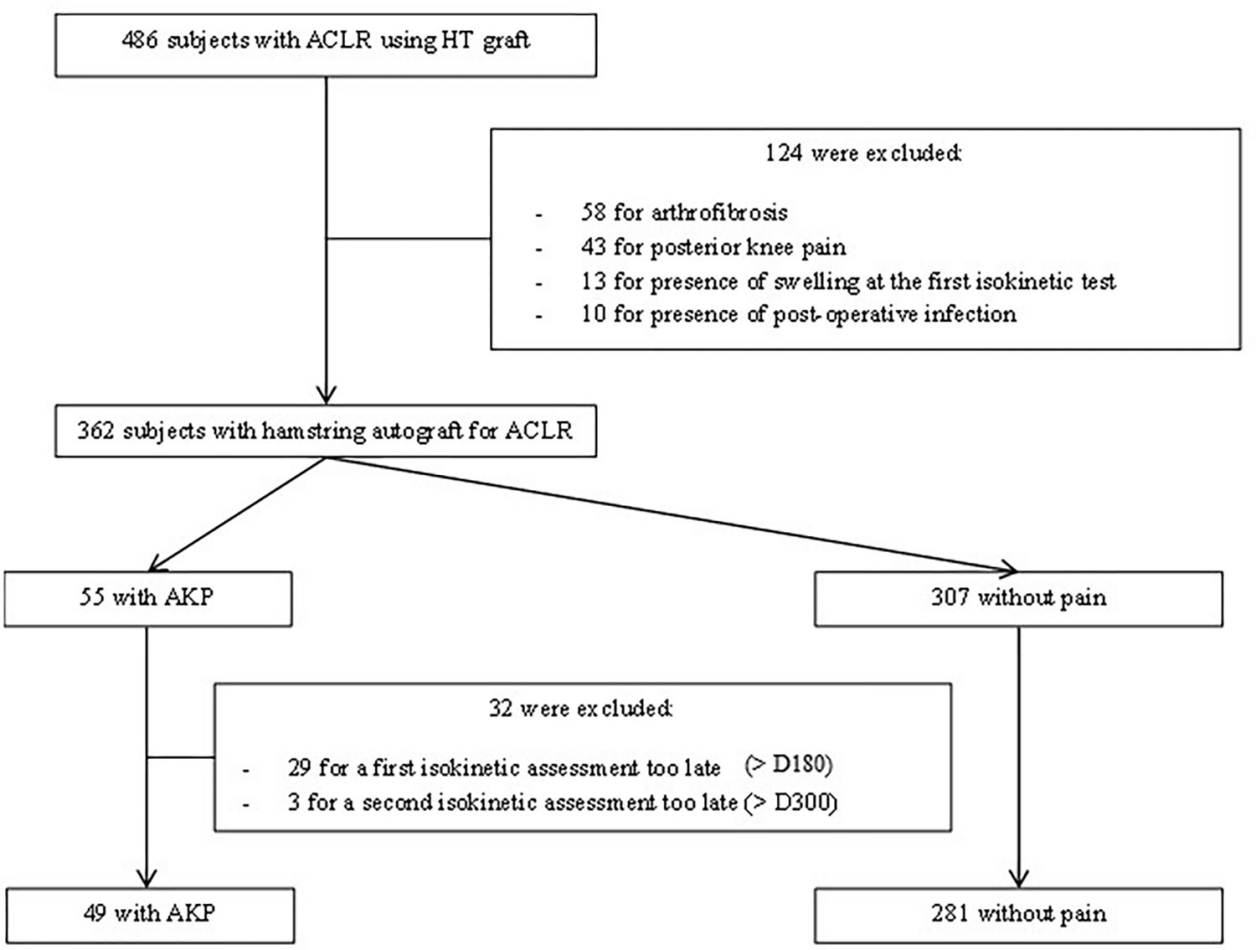
Flowchart of the recruitment and the selection of the participants. Abbreviations: ACLR: Anterior cruciate ligament reconstruction; AKP: Anterior knee pain; HT; Hamstring tendon; D180: 180 days after surgery; D300: 300 days after surgery.

A total of 486 patients between 2015 and 2020 with primary ACLR using HT autograft (semitendinosus-gracilis (STSG) graft and (ST) semitendinosus graft) were considered for eligibility to participate in this cross-sectional observational study. Their follow-up was carried out in the sports medicine department of the Nantes University Hospital. Inclusion criteria were age between 18 and 50 years old with at least, two isokinetic assessments, the first before 180 days post-surgery and the second before 300 days post-surgery. Exclusion criteria were the presence at 4 months post-surgery of arthrofibrosis, posterior knee pain (PKP), swelling at the first isokinetic assessment, sensitive pain due to an injury of the saphenous nerve, pain around surgical equipment such as screws and post-operative infection. After screening, a total of 330 individuals out of the original 486, was assessed and separated into two groups at 4 months post-surgery: the first one with the presence of AKP (AKP+ group) and the second one without pain (AKP− group) (Fig 1). If the patient complained of peri or retro patellar pain during the follow-up consultation at 4 postoperative months, just before the knee strength isokinetic measurement, the patient was included in the AKP+ group [7]. The functional consequences of AKP were defined by the Lysholm Score at the same time.

Each participant gave his written consent. The analysis of the data was carried out after anonymization. The local ethics committee (Comité Nantais d’Ethique en Médecine du Sport) under ethical committee registration CNEMS-2021-003 approved the study, which was declared to the Research Department of the University Hospital. The study was in accordance with the declaration of Helsinki [26].

### Surgical procedure

ACLR were performed with standardized arthroscopic procedure by different experienced surgeons, all of them performing ACLR on a regular basis.

First, an arthroscopic diagnosis was performed, including assessment of meniscus injuries that may lead to meniscal surgery, depending on the type and location of the tear. Then, HT autograft (STSG or four or three strand of ST) was harvested through a longitudinal incision over the pes anserinus insertion. The graft diameter measured between 7 and 9 mm. Tibial and femoral tunnels were drilled with out-in or in-out procedures, and HT autograft was fixed using endo-button (TighttRope® Arthrex, Naples, Etats-Unis) or screw for femoral fixation and screw for tibial fixation (Biosure® Arthrex, Naples, Etats-Unis). Sometimes a Tape Locking Screw was used (TLS, FH Orthopedics, Heimsbrunn, France).

### Rehabilitation protocol

As previously reported, early rehabilitation was performed [27]:

- Treatment of knee swelling, oedema and pain: compression, icing and non-steroidal anti-inflammatory drugs until complete swelling and heat resorption.
- Limitation of standing and walking to avoid pain and knee swelling.
- Full weight-bearing with crutches.
- Use of a total extension knee brace to achieve a full and active knee extension.
- Progressive recovery of knee range of motion, with special care for preserving full active knee extension all along the process.

Mid-term rehabilitation included:

- Stopping the use of crutches after obtaining a well-balanced gait thanks to proprioception exercises.
- Driving and return to sedentary professional activities, once walking without crutches has been validated.
- Authorisation to return to cycling when knee range of motion reached at least 0 to 120 degrees. A progressive protocol was given to every patient: only cycling was practiced 3 times a week, from 15-min sessions to 90-min sessions over a 2-month period, until the first isokinetic test.
- No return to other sports before the first isokinetic assessment was proposed.

### Isokinetic and functional assessments

Patients performed a systematic assessment at about 4 months post-surgery (and before 180 days) for the first isokinetic evaluation. A second assessment was performed at about 7 months post-surgery (before the 300^th^ postoperative day). The isokinetic assessment was performed by the same Physical Medicine and Rehabilitation (PMR) doctor as previously described [28–30]. Each participant warmed up for 10 minutes on a cycle ergometer. Then, the patient was installed on a Humac^®^ isokinetic dynamometer (Medimex, Sainte-Foy-lès-Lyon, France) in accordance with a standardized position maintained by belts stabilizing the trunk and the pelvis with a hip flexion of 85°, and an alignment between the mechanical axis of the dynanometer and the lateral condyle of the homolateral knee to the hip. The patient performed three submaximal and then, two maximal movements to get used to the required movement. Apart from the acceleration and deceleration phases, this technique made it possible to obtain a muscle contraction with a predefined and stable angular speed. As required, the knee joint range of motion varied from 0° of extension to 100° of flexion. The force torque was corrected by gravity in accordance with the constructor recommendations. The device was recalibrated monthly. The isokinetic assessment consisted of three repetitions at an angular velocity of 60°/s, followed by a recovery period of 30 seconds and then, five repetitions at 180°/s. Visual support and oral encouragement were provided throughout the assessment. The limb symmetry index (LSI) for the knee extensors or flexors was the principal parameter of the study. It was calculated according to the formulae: (peak torque of the operated knee report to the peak torque of the non-operated knee) x 100 [31].

Between the two isokinetic test sessions, the physician gave advice to the subject according to the results of the first isokinetic test. In case of an important quadricipital strength deficit (LSI < 60%), it was suggested to remain at rest or to continue cycling and not to start running. In case of quadricipital LSI ≥ 60%, return to running was allowed [32]. The reliability of the quadriceps and hamstring LSI was considered good to excellent (ICC: 0.78–0.98) [33].

The second parameters of interest were the Lysholm score at 4 and 7 months post-surgery, and the one leg hop test performed during the consultation for the second isokinetic evaluation, i.e., 7 months postoperatively. The practice of sport and the sport level before ACLR was determined using the Tegner activity-level scale [34], which determine a score ranging from 0 (person with a disability) to 10 (sports activity at a national or international level) [35]. This scale has been described valid and reliable for the assessment of the activity level before and after ACLR [36].

The Lysholm score measures the functional impact in terms of disabilities and activity limitations. Twenty-five points out of 100 describe the pain during activity (no pain = 25 points; light or intermittent pain during vigorous activities = 20 points; marked pain during mild-effort exercises = 15 points; marked pain during walking more than 2 km = 10 points and less than 2 km = 5 points and constant pain = 0 point) [37]. Eight items are reported with a maximal score of 100 points [37,38]. A global score of less than 65 points was considered low, while a score of more than 84 meant a high score. The Lysholm score has also been described reliable and valid for functional assessment after ACLR [36].

The one leg hop test was measured using a tape meter with a precision of 1 cm. Each player had to jump as far as possible using one leg, beginning with the non-operated side. The jump was validated if the balance on arrival was maintained for two seconds. The hop LSI was calculated as the isokinetic LSI. Hop tests are considered reliable and valid to measure a performance-based outcome [39].

### Statistical analysis

Data analysis was performed with SPSS 23.0^®^ software (Armonk, NY, USA). Quantitative parameters were presented as means and standard deviations while qualitative parameters were expressed as absolute values and percentages. The normality of the tested parameters was assessed by a Kolmogorov-Smirnov test.

At baseline, to compare the two groups (AKP+ and AKP–), either a t-test was performed after having performed a Levene’s test for the comparison of variances in case of quantitative variables, or a χ2 test in case of qualitative variables. The time effect between the two isokinetic tests was measured by comparing the AKP+ and AKP–groups using an analysis of variance for repeated measures (2 times x 2 groups). A generalized linear multivariate (GLM) model analysis was carried out to assess the weight of potential inter-subject explanatory parameters of the evolution of the knee strength LSI. The assumption of sphericity was assessed and corrected using the epsilon of Greenhouse-Geisser. Paired comparisons were performed with Bonferroni test. Effect sizes were assessed by partial eta squared η2 which were defined as trivial, small, moderate, and large for values η2 <0.01, > 0.01, > 0.06, > 0.14, respectively [40]. The alpha level of statistical significance was set at p < 0.05.

## Results

### Population

**Fig 2:**
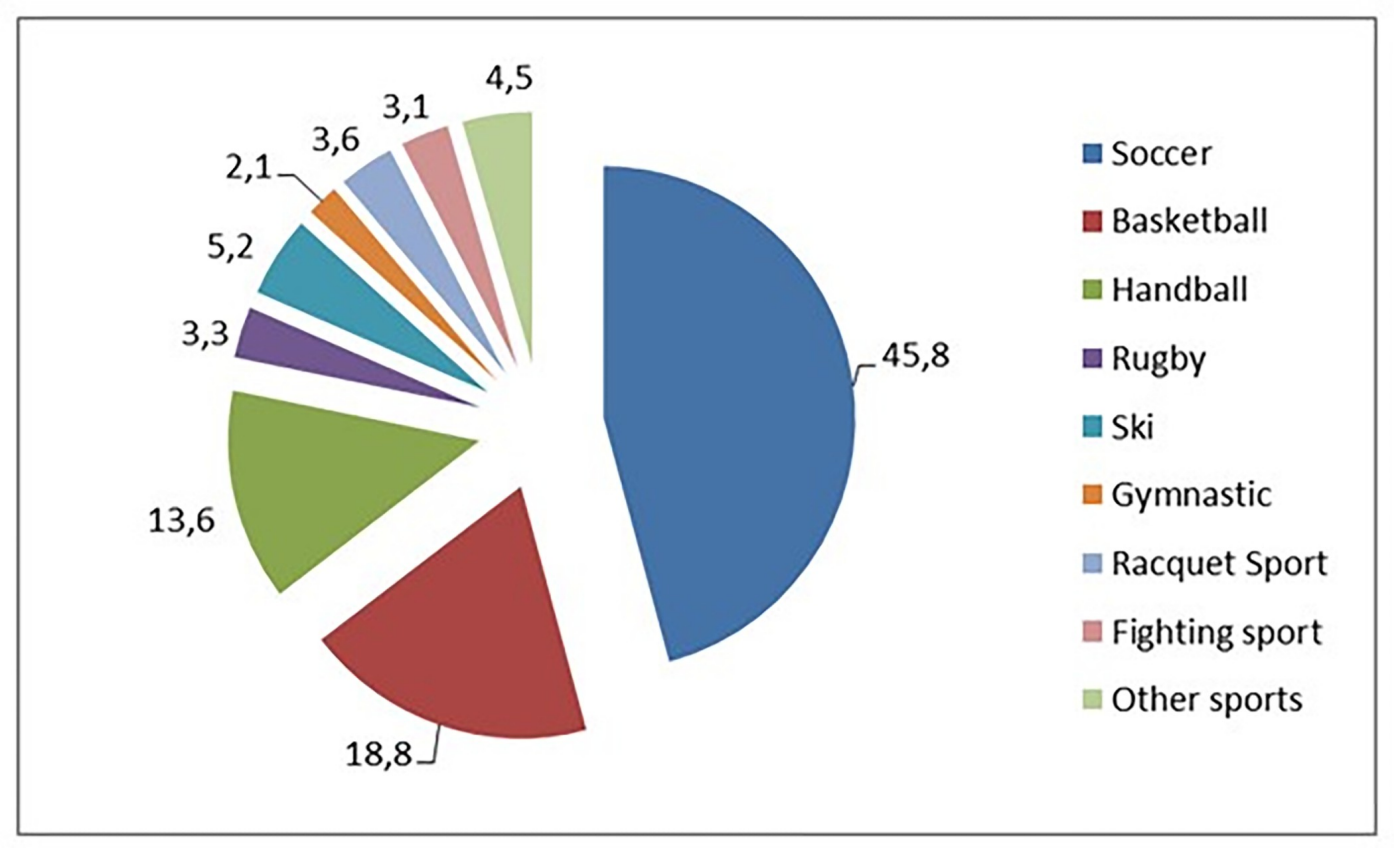
Sport practice before ACLR (%).

A total of 330 subjects (age of 26.4 ± 7 years old; weight of 74.0 ± 15 kg; height of 174.0 ± 9 cm) were evaluated. There were mostly men (71.2%). The most practiced sport before ACLR was soccer in 45.8% of cases (Fig 2).

The Tegner level sport before ACLR was 7–8 (56.6%), which corresponds to practice competitive sports, 6.7% of the population competed at an elite level, i.e. Tegner 10. ACLR was performed in 75% of cases by STSG and in 25% by ST. Lateral extra articular tenodesis and meniscal surgery were associated with ACLR in12.4% and 24.2% of cases, respectively. Continuous physiotherapy was performed in 28.5% of subjects at the time of the first isokinetic assessment (Table 1).

**Table 1:** Description and comparison of the subjects with ACLR according to the presence or the absence of AKP.

	ACLR (n = 330)	AKP+ group (n = 49)	AKP− group (n = 281)	*p*
Age (years)	26.4 ± 7	29.7 ± 7.7	25.8 ± 6.8	< 0.0001
Weight (Kg)	74.0 ± 15	76.1 ± 15.6	73.6 ± 14.8	0.28
Height (cm)	174.0 ± 9	174.5 ± 9.5	173.9 ± 8.9	0.79
Sex				0.70*
Male	235 (71.2%)	36 (73.5%)	199 (70.8%)	
Female	95 (28.8%)	13 (26.5%)	82 (29.2%)	
Tegner before surgery				<0.05*
5	35 (10.6%)	14 (28.6%) _a_	21 (7.5%) _b_	
6	54 (16.4%)	7 (14.3%) _a_	47 (16.7%) _a_	
7	110 (33.3%)	12 (24.5%) _a_	98 (34.9%) _a_	
8	77 (23.3%)	8 (16.3%) _a_	69 (24.5%) _a_	
9	32 (9.7%)	5 (10.2%) _a_	27 (9.6%) _a_	
10	22 (6.7%)	3 (6.1%) _a_	19 (6.8%) _a_	
Surgery				<0.05*
STSG	246 (74.5%)	31 (63.3%) _a_	215 (76.5%) _b_	
ST	84 (25.5%)	18 (36.7%) _a_	66 (23.5%) _b_	
Lateral extra articular tenodesis	41 (12.4%)	6 (12.2%)	35 (12.4%)	0.96*
Meniscal surgery	80 (24.2%)	13 (26.5%)	67 (23.8%)	0.96*
In-centre rehabilitation	198 (60%)	31 (63.3%)	167 (59.4%)	0.61*
Continuous physiotherapy	94 (28.5%)	13 (26.5%)	81 (28.8%)	0.74*
Retraining program				< 0.05*
Resting	24 (7.3%)	14 (28.6%) _a_	10 (3.6%) _b_	
Cycling	76 (23.0%)	17 (34.7%) _a_	59 (21.0%) _b_	
Running	230 (70.7%)	18 (36.7%) _a_	212 (75.4%) _b_	

Abbreviations: ACLR: Anterior Cruciate Ligament Reconstruction; AKP: Anterior Knee Pain; STSG: Semitendinosus-gracilis graft; ST: Semitendinosus graft

*: χ^2^- test; a: No difference between AKP+ and AKP–groups; b: Difference between AKP+ and AKP–groups.

The prevalence of AKP was 14.8% (49/330). No difference was found for height and weight. There was no difference in terms of number of lateral extra articular tenodesis or meniscal surgery but also in terms of post-surgery rehabilitation and continuous physiotherapy. The AKP+ group was significantly older than the AKP–group (29.7 ± 7.7 vs. 25.8 ± 6.8; *p* < 0.0001). Concerning the level of sport before surgery, no difference was shown excepted for level 5 (28.6% for the AKP+ group vs 7.5% for the AKP–group; p < 0.001). The AKP+ group had statistically less STSG (63.3% vs 6.5%; *p* < 0.05) and more SG (36.7% vs 23.5%; *p* < 0.05) than the AKP–group. Finally, the AKP+ group ran less (36.7% vs 75.4%; *p* < 0.0001), cycled more (34.7% vs 21.0%; p < 0.0001) and were more at rest (28.6% vs 3.6%; *p* < 0.0001 respectively) than the AKP–group (Table 1).

### Muscular and functional parameters at the first and second isokinetic tests after surgery

For the overall population, the first and the second isokinetic measurements were carried out at 124 ± 20 days and 210 ± 34 days, respectively.

At the first post-surgical isokinetic evaluation, the quadriceps LSI was significantly lower in the AKP+ group than in the AKP–group both at 60°/s and 180°/s. Hamstring LSI at 60°/s and Lysholm score were also lower in the AKP+ group than in the AKP–group (Table 2).

**Table 2:** Comparison of the different evaluations according to the presence or the absence of AKP.

		AKP+ group (n = 49)	AKP− group (n = 281)	*p*
**Time of the first isokinetic test (days)**		123 ± 16	125 ± 20	0.47
**Time of the second isokinetic test (days)**		206 ± 28	210 ± 35	0.40
**First isokinetic assessment**	Lysholm score	85.4 ± 9.4	96 ± 7.2	< 0.05
	Q60 LSI (%)	55.7 ± 17.9	72 ± 15.3	< 0.05
	Q180 LSI (%)	66.3 ± 17.6	78.3 ± 13.7	< 0.05
	H60 LSI (%)	79.1 ± 15.8	84.4 ± 12.7	< 0.05
	H180 LSI (%)	83.6 ± 18.5	85.9 ± 14.5	0.32
**Second isokinetic assessment**	Lysholm score	97.4 ± 6.2	97.5 ± 5.9	0.96
	Q60 LSI (%)	80.9 ± 16.1	80.4 ± 14.9	0.84
	Q180 LSI (%)	85.5 ± 18.7	84.2 ± 13.3	0.56
	H60 LSI (%)	89.5 ± 12.1	91.2 ± 47.2	0.80
	H180 LSI (%)	89.4 ± 13.1	89.6 ± 13.4	0.69
	Hop LSI (%)	93.1 ± 6.3	91.3 ± 7	0.17

Abbreviations: AKP: Anterior Knee Pain; Q60/180 LSI: Quadriceps Limb Symmetric Index at 60/180°/s of isokinetic angular speed; H60/180 LSI: Hamstring Limb Symmetric Index at 60/180°/s of isokinetic angular speed.

At the second post-surgical isokinetic assessment, no significant difference was found for the knee strength LSI, the one leg hop LSI or the Lysholm score (Table 2).

### Multivariate analysis

The three parameters which were different between the two groups, i.e., age, surgical procedure type and retraining program (Table 1) at baseline were introduced as inter-subject parameters in the multivariate analysis. Despite this statistical difference, for the level 5 of the Tegner score, this parameter was not included in the multivariate analysis due to the small sample and the lack of clinical relevance. We also analysed the effects of time and the presence of the AKP.

**Fig 3:**
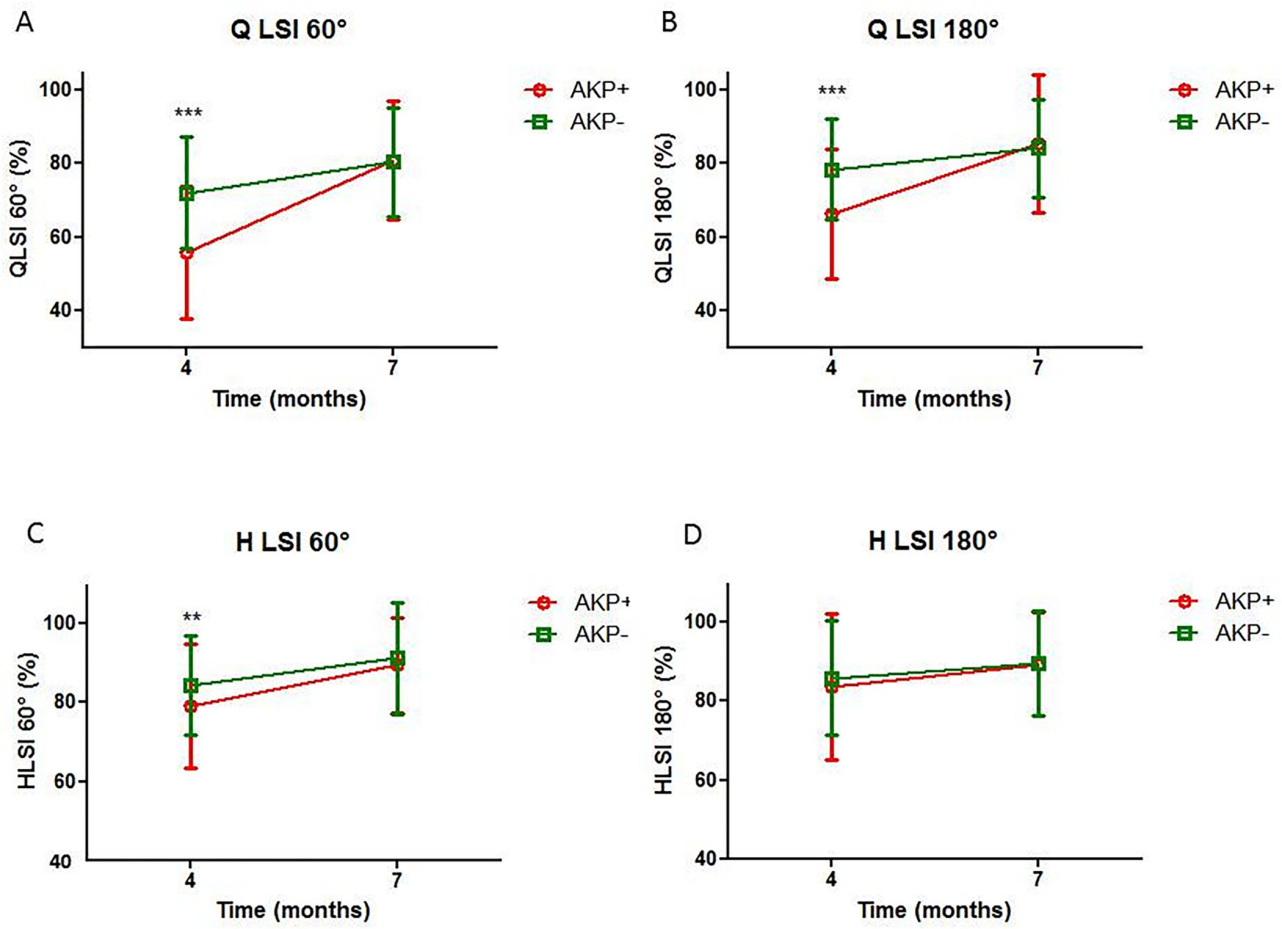
Evolution of the isokinetic parameters between the first and second isokinetic post-surgical tests according to the presence of anterior knee pain. Abbreviations: AKP: Anterior knee pain; Q60: Quadriceps at 60°/sec; Q180: Quadriceps at 180°/sec; H60: Hamstring at 60°/sec; H180: Hamstring at 180°/sec; LSI: Limb symmetry index.

Concerning the quadriceps LSI at 60°/s (Q60LSI), we found a large effect of the time (F(1.328) = 100.4; p < 0.05; η2: 0.23) and a moderate effect of the AKP (η2: 0.07). For the AKP+ group, the mean value in the first and second isokinetic tests was 55.6 ± 0.2% and 81.1 ± 0.2% respectively and, for the AKP–group, 72.0 ± 0.2% and 80.3 ± 0.1% respectively. Age, surgical procedure, and retraining program had a trivial or small effect (η2: 0.09; η2: 0.004; η2: 0.016 respectively) (Fig 3A).

Concerning the quadriceps LSI at 180°/sec (Q180LSI), the duration was also the only parameter with a large effect (η2: 0.16). The other parameters, i.e., presence of AKP, age, surgical procedure and retraining had a trivial or small effect (η2: 0.05; η2: 0.09; η2: 0.001; η2: 0.01 respectively) (Fig 3B).

Concerning the hamstring at LSI 60°/s (H60LSI) (Fig 3C) and the hamstring LSI at 180°/s (H180LSI) (Fig 3D), all the variables had a trivial or small effect (η2: 0.02 and 0.003 respectively for time effect; η2: 0.001 and 0.002 respectively for the presence of AKP; η2: 0.03 and 0.05 respectively for age; η2: 0.002 and 0.003 respectively for surgical procedure; η2: 0.001 and 0.011 respectively for retraining).

Concerning the Lysholm score, the presence of AKP was the only variable with a moderate effect (η2: 0.12). The other variables had a trivial or small effect (η2: 0.08 for time effect; η2: 0.08 for age; η2: 0.001 for surgical procedure; η2: 0.009 for the retraining).

## Discussion

The prevalence of AKP in our population with ACLR using HT graft was 14.8%. Subjects with AKP presented lower quadriceps and hamstring LSI and a lower Lysholm score at the first isokinetic test (about 4 months post-surgery) than subjects without AKP. However, the difference between the two groups did not persist at the second isokinetic test (about 210 days or 7 months post-surgery). No difference was shown on the one leg hop test at that same postoperative duration. For the evolution of the quadriceps LSI between the two isokinetic tests, a time effect existed.

In the literature, the rates of AKP post ACLR remained variable according to the studies. In a review of 2011 [2], having a PT graft generated a relative risk of 1.45 of developing AKP compared to having a HT graft. In comparative studies, Sajovic et al. described a prevalence of AKP of 17% for their HT group and of 19% for their PT group [11], and Rousseau et al. found AKP in 12.6% of subjects having a HT graft and 23.3% having a PT graft [5]. Thus, our result of 14.8% was in accordance with the literature concerning AKP after ACLR using HT graft.

Yet, the definition of AKP remains unclear [2,8,41]. No consensus exists concerning the definition, and the term of AKP is often used as a synonym of patellofemoral pain syndrome (PFPS). For example, AKP may be explained by intra-articular knee pain (such as chondromalacia patella, patellofemoral dysfunction, etc.), or a knee tendinopathy (such as patellar tendinopathy) [8,41,42]. Moreover, the spontaneous anterior knee symptoms may be increased by knee mobilisation (kneeling test, squatting, ascending or descending stairs, sitting position, etc.) [2,8,11,43]. When AKP is assimilated to PFPS in the literature, a clinical flowchart is frequently proposed with, as a starting point, a distinction made between a muscular defect or a misalignment [42]. Furthermore, five questions are asked about the description of symptoms, lower limb alignment, patella position, muscles and soft tissues conditions and knee function [42]. Considering that there is no universal PFPS, personalized diagnosis makes it possible to adapt management to each situation and to propose the most appropriate treatment [42]. Similarly, to help make the diagnosis of AKP without a surgical history, a checklist has been proposed in 2017 [8]. It considered both subjective information i.e., age, localisation of pain (front of knee or retro-patella), chronicity (duration longer than three months) and aggravating factors and objective information i.e., reproduction of symptoms during squatting, kneeling, or climbing stairs and presence of pain during some movements [8]. It added some exclusion criteria such as a previous lower limb surgery or a history of trauma [8]. Thus, all these elements seem to be interesting to help the clinician to accurately diagnose AKP post ACLR. Yet, the deficit of range of motion (ROM) was not mentioned in the different criteria, whereas both pain and ROM deficit may correspond to another complication such as arthrofibrosis in case of knee surgery [1,14,44]. Indeed, Marques et al. showed that AKP was 5.3 times more present with a loose knee extension than with a complete knee ROM [14]. To avoid misunderstanding concerning the diagnosis of AKP in our study, all our patients had full knee range of motion, thus excluding differential diagnoses such as arthrofibrosis or Cyclops syndrome [45,46].

ACLR (without complication) is responsible for a decrease of the maximal voluntary quadriceps isometric contraction, due to a neuronal central inhibition [16]. Park et al. showed a pain-independent action on the decrease of both voluntary and involuntary activations of the quadriceps, by simulating AKP through an injection of sterile hypertonic saline [19]. Likewise, after ACLR using HT graft, patients who presented AKP had a decrease in knee extensors isokinetic strength compared to those without AKP [47]. In our study, we also found a significant decrease in the quadriceps LSI for the subjects with AKP at 4 months after surgery. At the second isokinetic test (7 months after surgery), the quadriceps LSI and the hamstring LSI were comparable between the groups (AKP+ vs AKP–) at 80% and 90% respectively. According to the literature, the flexors and extensors LSI are of 90% at 2 years in case of HT graft [23,48,49]. So, at 7 months post-surgery, the quadriceps of the operated knee showed a persistent strength deficit of 20%. This AMI has been described as a possible protective mechanism to avoid overloading the knee joint in case of damage, especially in case of gonalgia, swelling or inflammation by stimulating the joint receptors [18]. The pathogenicity is complex and appears to involve both spinal and supra-spinal reflex pathways [50]. In our study, the AKP+ group were significantly older than the AKP- group, and in the literature it has already been described that some confounding factors, such as age, may contribute to explain lower isokinetic test results [16]. However, this parameter did not show any influence when performing the multivariate analysis. At 7 months post-surgery, the absence of difference in all the isokinetic strength parameters of both the quadriceps and the hamstrings can be partly explained by the natural evolution due to the effect of time. Indeed, the majority of AKP and AMI spontaneously disappear, resulting in an estimated prevalence of AKP of only 2.7% two years after surgery [5].

We found a lower Lysholm score in the AKP+ group at 4 months post-surgery, with no persistent difference at 7 months post-surgery. No difference was also found for the one leg hop test measured only at 7 months post-surgery. A lower score in lower-extremity functional outcomes has been described for all types of knee pathology and, in particular, AKP without surgical history and ACLR without complication [16]. Our results are consistent with those described by Dauty et al. [47] who found, in 2006, a lower Lysholm score in the AKP+ group at 4 months post-surgery with no difference at 7 months post-surgery. The one leg hop test is interesting for assessing functional muscle performance in patients with AKP [42]. In our study, the one leg hop test LSI at 7 months post-surgery ranged from 91.3% to 93.1%, with no difference between the AKP+ and AKP–groups. This result was comparable with those of the one-leg hop test described in the literature (> 90%), which was consistent with good functional recovery [13,25,49].

We can also discuss the influence of the type of retraining (cycling, running or resting) to obtain comparable results in the two isokinetic tests. Similarly, the regular practice of sport found in most subjects may have an impact on rehabilitation care. Indeed, having a higher level of sport may favour a management in a specialised centre, with continuous physiotherapy for up to 4 months after surgery and with, perhaps, a greater motivation regarding rehabilitation. However, being treated in a rehabilitation centre did not seem to prevent the occurrence of AKP in this study. Similarly, the type of retraining and the high level of sport before surgery did not seem to impact the rate of AKP. However, after the result of the first isokinetic assessment, the type of retraining i.e., resting, cycling or running was allowed according to knee extensors LSI (i.e., LSI < 60% or LSI ≥ 60%) [32].

From a general point of view, to prevent AKP, rehabilitation should aim symmetrical extension between the operated knee and the contralateral side after surgery [44]. Controlling acute pain and swelling is part of the initial management, as is muscle strengthening throughout rehabilitation [9]. The assessment of the pain by the physiotherapist would allow an adaptation of the session with antalgic technics such as cryotherapy [9]. Furthermore, isokinetic rehabilitation seems to improve pain and knee function [7]. In addition, both eccentric and concentric modes of contraction can be used [51]. Warning the patient in advance that AKP is a possible complication whatever the type of graft, and that it will evolve favourably over time seems essential. Indeed, as in all chronic pain, the psychological state would influence the sensation of pain [24].

This study presented several limitations. Firstly, without a consensus on the definition of AKP after ACLR, there could be a bias in patient recruitment. However, we included patients with pain only in the retro or peri-patellar region as described in the literature [7] and we excluded patients with a deficit of range of motion to avoid arthrofibrosis or cyclop syndrome. Secondly, AKP was only characterized by its presence or absence during the patient’s interview using the Lysholm score. The use of other scores might have improved the accuracy of patient selection. In addition, our results may not be generalizable to females because of the large proportion of men in the study, especially since the number of patients with AKP remains modest despite the size of the cohort. This predominance of males in our cohort is certainly related to the follow-up of sports subjects who particularly practiced contact sports such as soccer or basketball before ACLR. This may represent a recruitment bias. The small number of women may have prevented an association between post-ACLR AKP and pre-existing patellofemoral syndrome, which is more frequently reported in young women. Yet, this study allowed the comparison of similar groups with systematic assessment at specific post-operative times used in the medical follow-up of ACLR using an HT procedure. Finally, the follow-up was relatively short. A longer follow-up would have been interesting to obtain more information such as the link between AKP and the return to sport at the same level, for example.

## Conclusion

The prevalence of AKP after ACLR using HT graft at 4 months post-surgery was not negligible with a rate of 14.8%. AKP appears to be a disabling complication due to the resulting muscle strength inhibition and its functional consequences. Isokinetic evaluation is very interesting to detect the consequences of AKP on muscle strength, especially at 4 months after surgery, in order to personalize the retraining. It also helps guide the return to physical activities. The lack of consensus on the definition of AKP, particularly after ACLR, contributes to the challenge of its diagnosis, prevention and treatment.
